# Intramedullary Nail and Plate Combination Technique for Peri-Implant Both-Bone Forearm Fractures

**DOI:** 10.7759/cureus.28828

**Published:** 2022-09-06

**Authors:** Robert C Jacobs, Michael W Schmitz, Marc F Swiontkowski

**Affiliations:** 1 Department of Orthopaedic Surgery, University of Minnesota, Minneapolis, USA

**Keywords:** both-bone forearm fractures, open reduction internal fixation, intramedullary nail, nail and plate combination, periprosthetic fractures

## Abstract

A 35-year-old female patient with cerebellar ataxia presented with a right periprosthetic both-bone forearm fracture after a ground-level fall. Her surgical history was significant for multiple both-bone forearm fractures treated by open reduction and internal fixation. Subsequent treatment with a combination of intramedullary nailing and plate fixation for each bone provided successful fracture union while allowing immediate return to weight-bearing and range of motion. This case report demonstrates that intramedullary nailing and plate fixation of both-bone forearm fractures provides complete protection of the radius and ulna in recurrent, peri-implant both-bone forearm fractures. This technique is a valuable treatment option in the setting of a patient at risk for recurrent injury of the forearm.

## Introduction

Forearm fractures are often referred to as being very common injuries, yet diaphyseal forearm fractures are much less common, with an incidence reported to be 10 times less frequent than distal radius fractures [1]. These fractures are even less common in adults, as children account for roughly four-fifths of forearm shaft fractures [2]. Both-bone diaphyseal forearm fractures often result from high-energy trauma as well as indirect trauma due to bending or torsional forces with axial loading [3]. Although both-bone forearm fractures differ from related injuries such as Galeazzi and Monteggia fractures, they often result in damage to the interosseous space causing severe instability of the forearm and hand [4]. Surgical management of these fractures is necessary and has been well documented in the literature, with the majority of cases managed operatively via open reduction and internal fixation (ORIF) [5-7]. Peri-implant fractures at the ends of forearm fixation plates have historically occurred at rates ranging between 1% and 3% [8]. Intramedullary nailing (IMN) in select patients is a viable alternative to ORIF with locking plates for the treatment of both-bone diaphyseal forearm fractures due to shorter operative time, smaller incisions, and minimal dissection at the fracture site [9,10].

In geriatric distal femur fractures, a combination nail and plate technique has been demonstrated to be effective in allowing early weight-bearing and providing quality range of motion [11]. The IMN and plate fixation technique has also been shown to be effective in treating peri-implant fractures of the femur as it provides protection and stability of the bone while allowing full weight-bearing [12,13]. However, there is a paucity of data supporting the use of this technique for diaphyseal both-bone forearm fractures. In the following case, we describe the use of the intramedullary nail and plate combination technique for both the radius and ulna in the treatment of a recurrent peri-implant both-bone forearm fracture. This novel approach allows for complete protection of both the radius and ulna alongside immediate weight-bearing with a preserved range of motion.

The patient was informed that data concerning the case would be submitted for publication, and she provided consent.

## Case presentation

**Figure 1:**
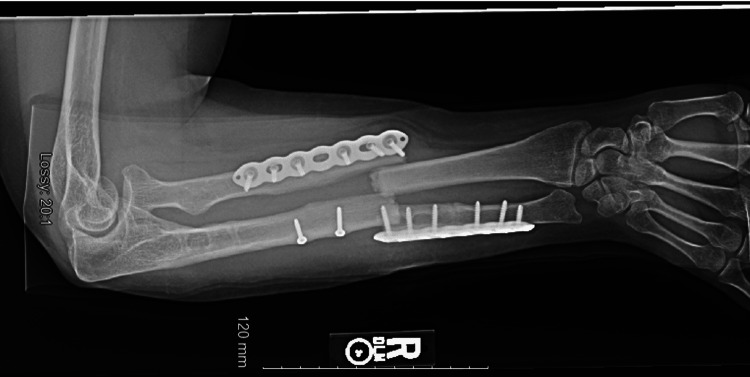
Anteroposterior forearm radiograph at the time of the injury. Independent screws and sclerosis can be seen in the metaphysis and proximal diaphysis of the ulna from her first fracture.

**Figure 2:**
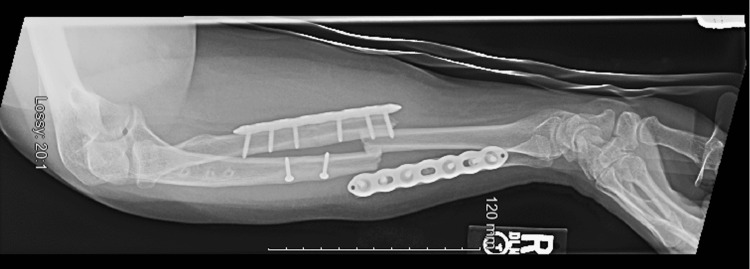
Lateral forearm radiograph at the time of the injury.

A 35-year-old female patient with a medical history significant for cerebellar ataxia presented after a ground-level fall in 2020. She sustained an isolated, right periprosthetic both-bone forearm fracture. Her surgical history was significant for a right forearm Monteggia fracture in 2009 which was treated successfully by ORIF of the right ulna using a compression plate. She subsequently sustained another right peri-implant both-bone forearm fracture in 2019 which was treated successfully with hardware removal and ORIF of the right radius and ulna using locking and compression plates. Figure 1 and Figure 2 demonstrate anteroposterior and lateral radiographs of the patient’s most recent peri-implant both-bone forearm fracture showing pronounced sclerosis in the proximal metaphysis of the ulna caused by her initial fracture in 2009. Given her history of repeated falls, we decided that protecting her entire radius and ulna would decrease the chance of future fractures requiring surgical fixation.

The patient underwent standard preoperative clearance prior to surgery. The radius was approached first through her previous volar Henry approach. The fracture site and hardware were exposed and removed. Provisional fracture reduction and fixation was achieved using a contoured 3.5 mm pediatric femur plate as this allowed for an appropriate length to be selected. Plate length was determined intraoperatively using a combination of preoperative and intraoperative radiographic measurements. The plate was then secured proximally and distally with a single eccentrically placed bicortical screw. A separate approach to the radial styloid was performed and care was taken to protect the branches of the radial sensory nerve and radial artery. A 3.0 mm flexible nail was then sized and passed retrograde to the level of the radial neck and buried within the radial styloid. The fracture reduction was confirmed, and the provisional screws were backed to unicortical. Compression of the transverse fracture was achieved through the plate using two screws immediately proximal and distal to the fracture. The unicortical screws were replaced with bicortical fixation. Lastly, the distal and proximal-most screws were placed. The incision was thoroughly irrigated and closed before addressing the ulna. The fixation process was repeated for the ulna using the previous subcutaneous ulnar incision. Again, the hardware was removed with subsequent provisional reduction and fixation using a 3.5 mm pediatric femoral plate. A 2.5 mm antegrade intramedullary flexible nail was placed through a separate incision overlying the proximal olecranon. Compression of the fracture was completed through the plate with balanced fixation across the entire plate length.

**Figure 3:**
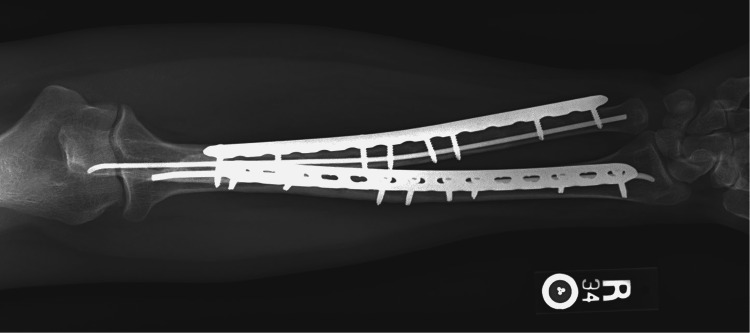
Anteroposterior forearm radiograph at 18 months after definitive surgery.

**Figure 4:**
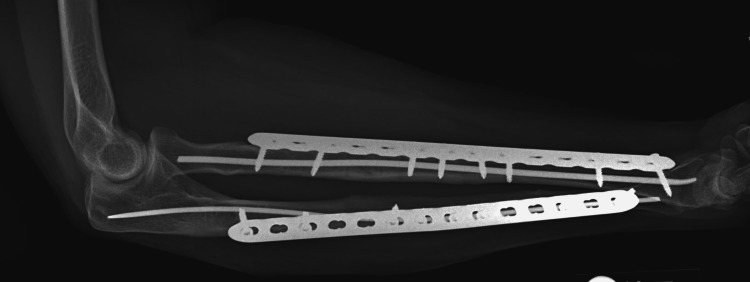
Lateral forearm radiograph at 18 months after definitive surgery.

The patient was allowed immediate range of motion as tolerated with no activity restrictions. Physical therapy to assist with the range of motion and gentle strengthening was allowed at six weeks. She was referred to a movement disorder clinic as well as an occupational therapist for her cerebellar ataxia. Her fractures had completely healed within five months with a final range of motion of 0-140° elbow flexion, 70° pronation, and 80° supination. Follow-up was completed 18 months after surgery, at which point there were no signs of recurrent bony injury of the operative extremity. Figure 3 and Figure 4 show anteroposterior and lateral radiographs at the time of the final follow-up.

## Discussion

Peri-implant both-bone forearm fractures are a very uncommon injury and can usually be treated with ORIF and hardware removal. Occasionally, the hardware can remain in situ and alternative approaches with overlapping fixation can be utilized. With this patient, we borrowed a fixation principle utilized in the distal femur for geriatric patients. In an effort to protect the entirety of both bones, we chose a nail and plate combination for the radius and ulna. The stiffer plates allow protection of the diaphysis while the flexible nails dissipate the stress risers into the proximal and distal metaphyses of both bones. The length of the plate is often determined intraoperatively using a combination of preoperative and intraoperative radiographic measurements. In this instance, we opted for plates that would provide coverage of the entire radial and ulnar diaphyses to decrease the patient’s risk of recurrent fracture.

We intentionally undersized the flexible nail by 0.5-1.0 mm for each bone. The diameter of the nail was balanced against the desire to achieve bicortical screw fixation as often as possible. Given the patient’s size and skeletal maturity, we were able to use a relatively large intramedullary nail in each bone. Screw placement occasionally required redirection around the nail, but in each instance, we were able to achieve quality bicortical fixation. We ensured quality bicortical screw fixation through a combination of tactile screw resistance during cortical penetration along with concurrent radiographic imaging to guarantee optimal implant stability.

The fractures were reduced and secured with provisional, eccentric bicortical fixation with one proximal and distal screw through the contoured plate. The screws were placed in this manner which served two purposes to help facilitate successful nail passage. First, eccentric placement allowed for easier nail passage. Second, bicortical fixation prevents fracture displacement of the transverse pattern when forceful mallet strikes are required to pass the tip of each nail past the sclerotic bone adjacent to the old screw tracts.

Each nail was sized and cut prior to passage. Care was taken to ensure that nail would span into each metaphysis when buried. Because the nails will be retained, we buried them completely within the bone to prevent back out. The paddle end of the nail tends to deflect off the sclerotic bone from the old screw tracts. Therefore, the stiffer cut end was passed in each case after using the abrasive portion of the drill collet to grind down the metal burrs which will prevent catching.

Variations of the above technique have been used successfully in the femur and tibia [14,15]; however, we are unaware of previously published reports of its use in the forearm. We are hopeful that this technique can be used by others in the future when select circumstances are present.

## Conclusions

Our work showcases well-known orthopedic trauma surgical approaches, though they have not been described previously in combination. We adopted a patient-specific surgical technique that has previously been used successfully in the treatment of certain femur fractures. This allowed us to safely address a complex, uncommon surgical problem and provide an excellent outcome.
